# The multiple autoantibodies in anti-GBM disease

**DOI:** 10.1093/ndt/gfaf211

**Published:** 2025-10-16

**Authors:** Maria Prendecki, Charles D Pusey

**Affiliations:** Centre for Inflammatory Disease, Department of Immunology and Inflammation, Imperial College London, Hammersmith Campus, London, UK; Centre for Inflammatory Disease, Department of Immunology and Inflammation, Imperial College London, Hammersmith Campus, London, UK

Anti-glomerular basement membrane (GBM) disease has long been viewed as a prototypic, organ-specific autoimmune disease defined by autoreactivity to a single dominant antigen. Patients develop an immune complex, small vessel vasculitis, usually with severe clinical features of rapidly progressive glomerulonephritis with crescentic glomerulonephritis on kidney biopsy and, in approximately half of cases, concurrent diffuse alveolar haemorrhage. The prognosis remains poor without urgent immunosuppression and plasmapheresis.

A key breakthrough in our understanding of the pathogenesis of anti-GBM disease was the identification of the ‘Goodpasture antigen’ within the noncollagenous (NC1) domain of the α3 chain of type IV collagen [α3(IV)NC1]. In the adult GBM, type IV collagen consists mainly of triple helical protomers of α3α4α5 chains, although α1α1α2 chains are also present. Two α3α4α5 protomers associate via their C-terminal domains to form the hexameric NC1 domain, the quaternary structure of which is stabilized by sulfilimine bonds. The pathogenic epitopes of α3(IV)NC1, E_A_ and E_B_, are normally cryptic within the quaternary structure of the NC1 domain and disruption of this structure is needed to reveal them [1].

It is now clear, however, that the immunology of anti-GBM disease is more complex. Studies using anti-GBM antibodies eluted from patients’ kidneys have shown that all bind to the α3 chain but some also bind to α5 and α4 chains [2]. Work using sera from anti-GBM patients showed that they may contain antibodies against all five α chains of type IV collagen in the GBM [3]. In ‘atypical’ anti-GBM disease linear IgG deposition is observed on kidney biopsy but standard serological testing using enzyme-linked immunosorbent assay (ELISA) for α3(IV)NC1 antibodies is negative [4]. These patients usually have a more indolent disease course without lung disease. A range of autoantibodies has been identified to cause disease in these cases, including atypical immunoglobulin isotypes such as immunoglobulin A (IgA) or IgG4, or those directed against non-classical epitopes such as other collagen α chains or non-collagenous basement membrane proteins. Immunoblotting using human GBM may detect some patients who are negative on standard immunoassays, however this test is not usually available for clinical use.

**Figure 1:**
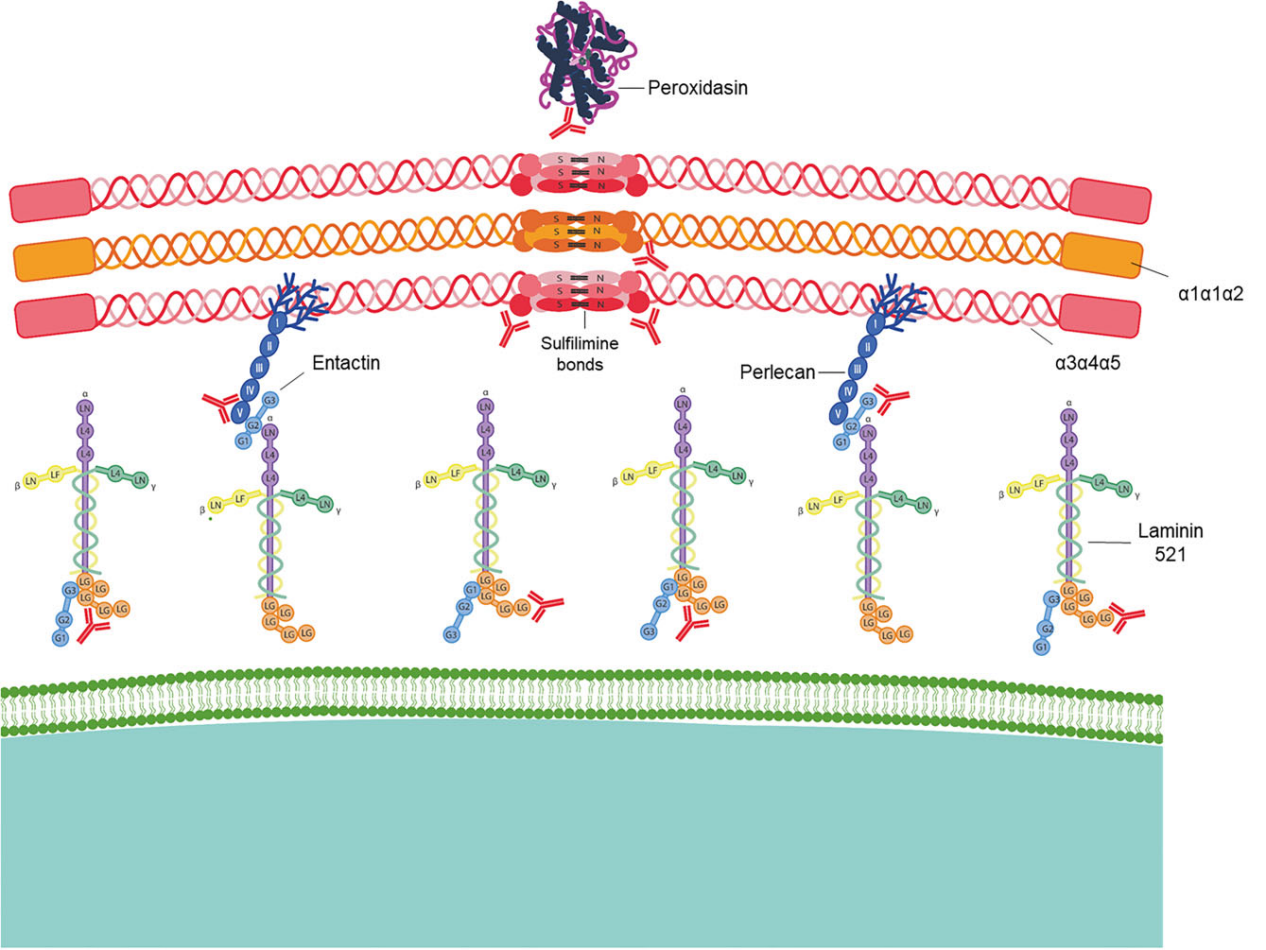
Autoantigens in anti-GBM disease. The predominant autoantigen in anti-GBM disease is the NC1 domain of α3(IV)NC1. Antibodies have also been detected against other α (IV) chains and against non-collagenous components of the GBM including laminin 521, nidogen-1 (entactin), peroxidasin and perlecan.

A growing list of non-collagenous autoantigens in anti-GBM disease has emerged in recent years (Fig. 1). Antibodies against laminin-521 (LM521), a major glycoprotein in basement membrane which is important in maintaining the glomerular filtration barrier, has been identified in around a third of patients with typical anti-GBM disease with an association with pulmonary haemorrhage [5]. Importantly, these antibodies are probably directly pathogenic. A case of atypical anti-GBM disease has been described in which circulating anti-LM-521 antibodies were detected in the absence of antibodies targeting the NC1 domains of α1–α5 of type IV collagen; titres of anti-LM-521 antibodies correlated with a falling creatinine as the patient responded to treatment [6]. Immunization of Wistar-Kyoto rats with recombinant human LM521 has confirmed pathogenicity, as this resulted in crescentic glomerulonephritis and pulmonary haemorrhage with linear IgG deposition on the GBM [6].

Peroxidasin is a haem peroxidase which is embedded in the basement membrane and plays a key role in the formation of sulfilimine bonds, which grant immune privilege to the pathogenic epitopes of the α3 chains of type IV collagen. Autoantibodies to peroxidasin have been identified in almost 50% of patients with anti-GBM disease, and 14% of patients with anti-myeloperoxidase (MPO) anti-neutrophil cytoplasm antibody-associated vasculitis (AAV) [7]. In anti-GBM disease, anti-peroxidasin antibodies may contribute to pathogenesis by inhibiting sulfilimine cross-linking, revealing pathogenic epitopes and leading to the development or binding of anti-GBM antibodies. They may also contribute to vascular injury by inhibiting peroxidasin function.

Entactin (now known as nidogen-1) is a sulfated glycoprotein which links type IV collagen with other basement membrane components. In case series of atypical anti-GBM disease it has been identified as a target autoantigen; however, this finding has not been replicated and whether it represents a clinically relevant autoantigen is not clear [8]. Despite this, the identification of entactin antibodies reinforces the concept of multiple antigenic targets in anti-GBM disease.

In this issue of *Nephrology Dialysis Transplantation*, Kuang *et al.* add another basement membrane component to this repertoire, identifying antibodies against perlecan, a large sulfate proteoglycan, in a proportion of cases [9]. Perlecan is an evolutionarily conserved, ubiquitous proteoglycan expressed in all basement membranes, cartilage and other mesenchymal tissues. Named for its ‘pearls on a string structure’ on electron microscopy, it has a modular structure with a large ∼470 kDa core protein consisting of five globular functional domains, and three additional N-terminal heparan sulfate side chains. Structurally, perlecan spans the basement membrane and anchors different membrane components including linking laminin to type IV collagen [10]. The N-terminal heparan sulfate chains can recruit complement factor H, suggesting a role in modulating alternative pathway activation. Although perlecan is not essential for maintaining the selectivity of the GBM filtration barrier, it may play a role in some settings; knock-in mice in which perlecan lacks heparan sulfate chains developed normally in homeostatic conditions but developed profound proteinuria in response to protein overload [11]. Knockout models highlight perlecan’s essential yet complex roles, with animals demonstrating broad phenotypes. Homozygous *Hspg2* knockout mice are embryonic or perinatal lethal due to either severe achondroplasia or abnormal basement membranes at sites of stress such as the cardiac outflow tract [12]. Kidney disease has not been reported as part of these genetic phenotypes.

In the current study, anti-perlecan antibodies were identified in approximately 20% of typical anti-GBM disease cases. The authors used murine perlecan, as the human protein was not available, which is a limitation of this work. Antibodies were predominantly IgG3 subclass and were associated with pulmonary haemorrhage, although this may be confounded by smoking history and the co-existence of anti-LM521 antibodies. In patients with atypical anti-GBM disease, defined as linear IgG along the GBM but no circulating α3(IV)NC1 antibodies on standard testing, 10% of patients had detectable anti-perlecan antibodies using ELISA, although immunoblotting was negative, suggesting low titres or different epitope specificity. Among patients with typical anti-GBM disease, the presence of anti-perlecan antibodies was frequently associated with anti-LM521 antibodies. There was a suggestion that patients who were ‘triple-positive’ for autoantibodies against all three GBM components exhibited a more severe disease phenotype, with a greater proportion of patients developing a composite end point of end-stage kidney disease or death compared with those positive for either anti-α3(IV)NC1 alone or the combination of anti-α3(IV)NC1 and anti-LM521. There was also an association between triple-positivity and the prevalence of lung haemorrhage, although this was lost on multivariable analysis, where anti-LM521 was identified as the independent risk factor. Intermolecular epitope spreading is one potential mechanistic explanation for the presence of antibodies against multiple components of the GBM. Increased clinical severity with a greater number of antigenic targets is in keeping with previous studies in anti-GBM disease, where a broader spectrum of antibodies against different collagen IV α chains was associated with more severe renal disease [3].

The authors did not identify anti-perlecan antibodies in a range of other glomerulonephritides. However, the identification of anti-perlecan antibodies in transplantation underscores that they are not uniquely associated with anti-GBM disease. Antibodies against a C-terminus fragment of perlecan, LG3, have been described in kidney transplant recipients, where they correlate with antibody-mediated rejection and adverse graft outcomes [13]. Endothelial injury may be the source of these antibodies, as the LG3 fragment has been shown to be released from apoptotic endothelial cells. Passive transfer experiments using anti-LG3 antibodies in mice identified that anti-LG3 antibodies enhanced renal dysfunction and classical pathway activation only when combined with an ischaemic insult, suggesting that these antibodies require a ‘second hit’ to exert pathogenicity [14].

In the present study, the authors were unable to directly assess the pathogenicity of anti-perlecan antibodies as all patients were also positive for anti-α3(IV)NC1 antibodies. Although cross-reactivity between autoantibodies against α3(IV)NC1, LM521 and perlecan was excluded, it was not possible to determine whether anti-perlecan antibodies arose primarily in disease or secondary to epitope spreading from glomerular injury. Given its biology, a pathogenic role for anti-perlecan antibodies in anti-GBM disease is mechanistically plausible; autoantibodies directed against perlecan may disrupt its structural bridging functions, impair growth factor signalling, and interfere with complement regulation. However, previous findings with LG3 antibodies suggest this pathogenicity may be restricted to situations where inflammation or a ‘second-hit’, such as anti-α3(IV)NC1 antibody–induced injury, is also present. In contrast, in early studies, passive transfer of antibodies against heparan sulfate proteoglycans was reported to induce GBM abnormalities and proteinuria; however, these antibodies may target other proteoglycans, such as agrin, in addition to perlecan [15]. A ‘second-hit’ hypothesis could explain the co-association between smoking, anti-perlecan antibodies and lung haemorrhage, whereby anti-perlecan antibodies may arise due to endothelial injury induced by smoking. When exposed to the inflammatory milieu induced by anti-GBM disease this could result in enhanced complement activation, leading to lung haemorrhage. Further work is required to confirm the presence of anti-perlecan antibodies in other populations of anti-GBM patients, to define their epitope specificity and to investigate their pathogenicity—perhaps using animal models.

The recognition of multiple GBM autoantigens has several potential clinical implications. Current clinical assays detect only α3(IV)NC1 antibodies and the incorporation of other α chains, laminin, perlecan and possibly peroxidasin into serological testing may enhance sensitivity, particularly in atypical cases. Identification of patients with antibodies against multiple antigens may allow earlier recognition of high-risk disease enabling informed treatment decisions. Beyond anti-GBM disease, the detection of GBM component antibodies such as perlecan and peroxidasin, in transplantation and AAV respectively, raises the possibility that basement membrane antibody panels could have utility across a spectrum of renal and pulmonary immune-mediated disorders.

In summary, anti-GBM disease was once regarded as the prototype of a single-antigen autoimmune disorder targeting α3(IV)NC1. However, there is now a growing repertoire of antibodies directed against various α chains of type IV collagen, LM521, peroxidasin, entactin and now perlecan. Future research is needed to identify the sequence of epitope spreading and the clinical impact of multiple autoantibodies in anti-GBM disease. However, the detection of antibodies to α3(IV)NC1 remains the most important defining feature of anti-GBM disease at present.
